# Cyclic Imines and Their Salts as Universal Precursors in the Synthesis of Nitrogen-Containing Alkaloids

**DOI:** 10.3390/ijms26010288

**Published:** 2024-12-31

**Authors:** Andrey Smolobochkin, Almir Gazizov, Nikita Sidlyaruk, Nurgali Akylbekov, Rakhmetulla Zhapparbergenov, Alexander Burilov

**Affiliations:** 1 Arbuzov Institute of Organic and Physical Chemistry, FRC Kazan Scientific Center, Russian Academy of Sciences, Arbuzov Str., 8, Kazan 420088, Russia; agazizov@iopc.ru (A.G.); chem@sidlyaruk.ru (N.S.); burilov@iopc.ru (A.B.); 2 Laboratory of Engineering Profile, Department of Engineering Technology, Korkyt Ata Kyzylorda University, Aiteke bi Str., 29A, Kyzylorda 120014, Kazakhstan; ulagat-91@mail.ru

**Keywords:** alkaloids, cyclic imines, *N*-heterocycles, natural products

## Abstract

Alkaloids are predominantly nitrogen-containing heterocyclic compounds that are usually isolated from plants, and sometimes from insects or animals. Alkaloids are one of the most important types of natural products due to their diverse biological activities and potential applications in modern medicine. Cyclic imines were chosen as starting compounds for the synthesis of alkaloids due to their high synthetic potential. Thus, this review summarizes the achievements in the synthesis of various alkaloids from cyclic imines, paying special attention to stereoselective methods used for their preparation. Information on the biological activity of some alkaloids, their application and occurrence in natural objects is presented. Synthesis methods are classified based on the type of alkaloid obtained.

## 1. Introduction

Alkaloids have long been known for their biological activity [1,2,3,4,5]. The alkaloid content in plants is low as a rule, so it is often difficult to isolate this valuable product in sufficient quantities. In this case, the isolated alkaloid may contain various undesirable impurities [6]. In addition, the process of alkaloid isolation causes irreparable damage to the environment, since large numbers of plants and trees are destroyed. In some cases, such plants are on the verge of complete extinction [7]. In such instances, synthetic organic chemistry comes in useful. Currently, synthetic organic chemistry is being replenished with modern synthesis methods, which make it possible to obtain very complex target compounds.

This review article is devoted to methods used for the synthesis of various alkaloids based on cyclic imines. Cyclic imines are an important class of nitrogen-containing heterocyclic compounds that can act as precursors for the synthesis of alkaloids [8,9,10]. This is due to the fact that cyclic imines contain several reaction centers: a nucleophilic nitrogen atom and an imine bond with an electrophilic carbon atom. They are easy to synthesize [11,12,13].

**Figure 1:**
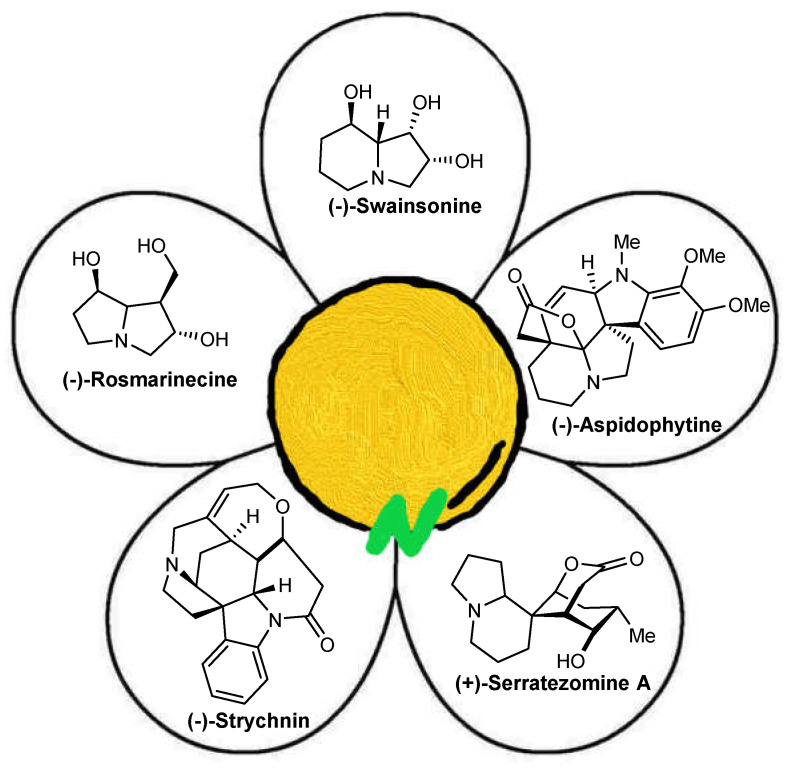
Example of alkaloids synthesized from imines.

The imine moiety can be reduced or oxidized and functionalized, which makes it a very valuable building block for alkaloid synthesis. Examples of valuable alkaloids synthesized from imines are shown in Figure 1. This figure shows the diversity of the structures of the resulting alkaloids based on cyclic imines. They include pyrrolizidine and indolizidine alkaloids with simple structures, as well as complex polycyclic alkaloids belonging to the classes Aspidosperma, Stephacidin, etc.

Currently, there are many publications in the literature devoted to the use of cyclic imines in the synthesis of alkaloids. This review is the first to summarize the scattered information on the synthesis of various classes of alkaloids. For convenience, we have grouped the material into sections according to the class of alkaloid.

## 2. Synthesis of Indolizidine Alkaloids

Indolizidine alkaloids are a broad class of natural compounds based on the indolizidine heterocycle [14,15] that have diverse biological activities. Swainsonine is a representative of indolizidine alkaloids, and was first isolated from the plant of *Swainsona canescens* species [16].

There are quite a lot of review articles related to the synthesis of Swainsonine [17,18,19,20], which indicates great interest in this alkaloid. Thus, it exhibits antitumor properties [21] and is an inhibitor of glycoprotein processing [22]. There is much evidence of Swainsonine poisoning in domestic animals that eat plants containing this alkaloid, which causes significant damage to livestock [23].

**Scheme 1:**
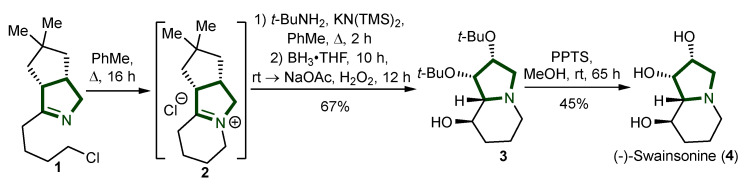
Synthesis of (-)-Swainsonine.

One of the methods used for the synthesis of Swainsonine (**4**) is the cyclization of imine **1** in boiling toluene, followed by treatment with *tert*-butylamine, potassium bis(trimethylsilyl)amide and borane–tetrahydrofuran, which leads to heterocycle **3** at 67% yield (Scheme 1). At the last stage, heterocycle **3** is treated with pyridinium *p*-toluenesulfonate (PPTS), which makes it possible to enantioselectively obtain (-)-Swainsonine (**4**) (>95% *ee*) [24].

**Scheme 2:**
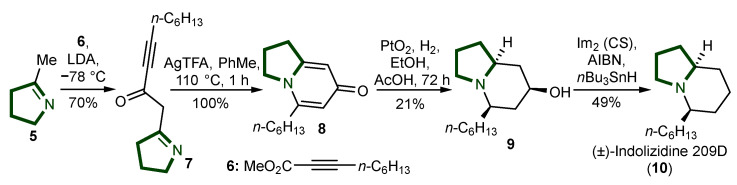
Synthesis of (±)-Indolizidine 209D.

In 2018, P. Unsworth and colleagues [25] reported efficient Ag(I)-catalyzed cyclization of ketamine **7** derived from 2-methyl-1-pyrroline **5**. It should be noted that the authors who created this method isolated the desired bicyclic product **8** in quantitative yield with further hydrogenation with hydrogen using platinum (IV) oxide as a catalyst. During the final stage, Barton–McCombie deoxygenation was carried out with the formation of (±)-Indolizidine 209D (**10**) (Scheme 2). Unfortunately, the absence of enantioselectivity renders this method less applicable to the synthesis of biologically relevant compounds. This alkaloid was first isolated from the skin of tropical frogs of the genus *Dendrobates* [26]. It has a number of pharmacological properties, including blocking the neuromuscular transmission [27].

**Scheme 3:**
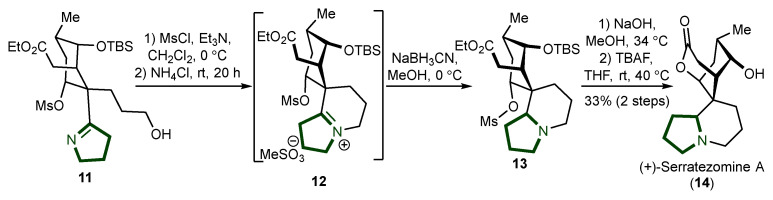
Synthesis of (+)-Serratezomine A.

Serratezomine A (**14**) was isolated from the club moss *Lycopodium serratum* relatively recently, in 2000 [28]. The use of an imine **11** as a starting compound made it possible to synthesize Serratezomine A quite efficiently (Scheme 3). Thus, cyclization of the imine **11** to mesylate **12** and subsequent reduction with sodium cyanoborohydride led to heterocycle **13**. Subsequent saponification and intramolecular cyclization resulted in Serratezomine A in a 33% yield [29].

**Scheme 4:**
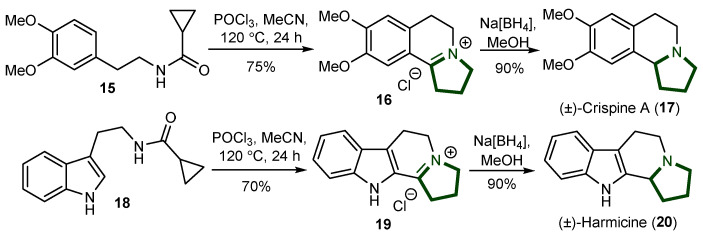
Synthesis of (±)-Crispine A and (±)-Harmicine.

Several review articles have been devoted to the synthesis of Crispine A (**17**) and Harmicine (**20**) [30,31]. This interest is due to the biological activity of these alkaloids [32,33]. It is very interesting that these alkaloids were synthesized before they were discovered in living objects. For example, Crispine A was isolated in 2002 from extracts of *Carduus crispus* [31]. It is quite simple to synthesize these alkaloids in almost quantitative yield by cyclization of amides **15** and **18** by an excess of phosphoryl chloride to heterocycles **16** and **19**, which could be easily reduced to products **17** and **20** with sodium borohydride in methanol [34] (Scheme 4). The above reactions can be carried out at gram scale; however, only racemic compounds were obtained.

**Scheme 5:**
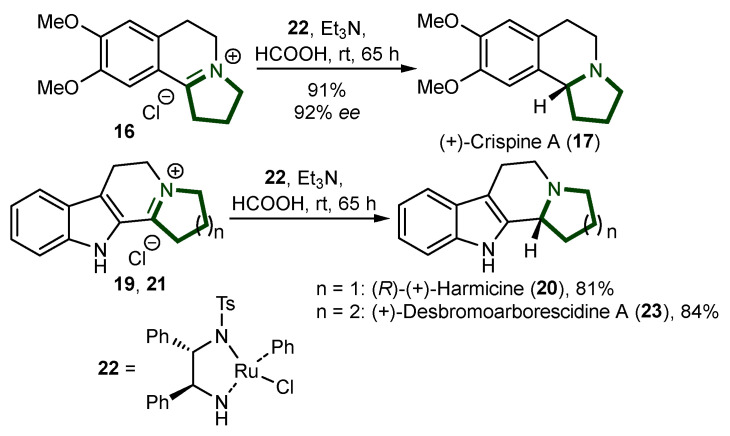
Synthesis of (+)-Crispine A, (+)-Harmicine and (+)-Desbromarborescidine.

In a series of works [35,36], a method for synthesizing (*R*)-(+)-Crispine A, (*R*)-(+)-Harmicine and (*R*)-(+)-Desbromarborescidine is described, based on stereoselective hydrogenation of iminium salts **16**, **19**, **21** in the presence of ruthenium catalyst (Scheme 5).

**Scheme 6:**
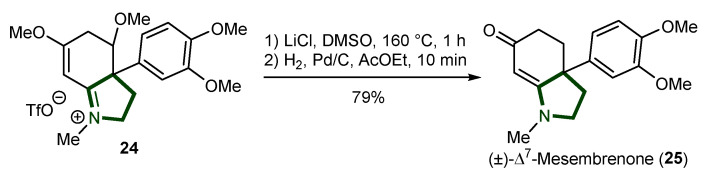
Synthesis of (±)-Δ7-Mesembrenon.

The alkaloid (±)-Δ7-Mesembrenone (**25**) could be isolated in one stage [37] from iminium salt **24** by demethylation and hydrogenation by Krapcho with a yield of 79%; this alkaloid exhibits various biological activities [38] (Scheme 6). The drawback of this method is the lack of enatioselectivity. Mesembrenone was found to be dominant alkaloid in the leaves of sprouting bulbs and in the flowers *Narcissus cv. Hawera* [39] and *Sceletium tortuosum* [40]. Mesembrine significantly contributes to the anxiolytic effect of Zembrin [41].

**Scheme 7:**
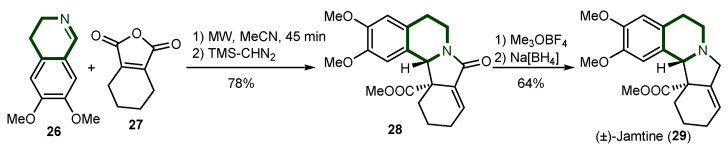
Synthesis of (±)-Jamtine.

**Scheme 8:**
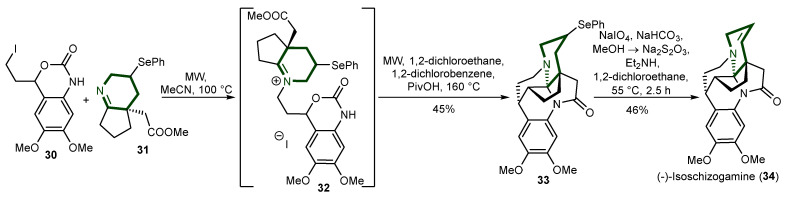
Synthesis of (-)-Isoschizogamine.

The authors of [42] described a short process for synthesizing (±)-Jamtine (**29**). The key step was the condensation of 6,7-dimethoxy-3,4-dihydroisoquinoline **26** and tetrahydrophthalic anhydride **27** by microwave activation, which occurred in good yield and high diastereomeric selectivity. Reducing the amide function resulted in (±)-Jamtine (Scheme 7). The alkaloid Jamtinine was isolated from whole plants of *Cocculus hirsutus* [43], which are renowned for their therapeutic properties [44,45,46].

(-)-Isoschizogamine (**34**) was first isolated in 1963 from the plant Schizozygia caffaeoides [47]. It exhibited antimicrobial and antifungal activity in micromolar concentrations [48]. Reacting compounds **30** and **31** under microwave irradiation resulted in iminium salt **32**, which was dissolved in 1,2-dichloroethane/1,2-dichlorobenzene and irradiated again (Scheme 8). Under these conditions, hexacyclic compound **33** was isolated in 45% yield as a mixture of two diastereomers (dr = 1:1). Oxidation of compound **33** to selenoxide followed by syn-elimination led to the production of (-)-Isoschizogamine [49].

## 3. Synthesis of Pyrrolizidine Alkaloids

Pyrrolizidine alkaloids are a group of compounds that predominantly originate from plants which contain a pyrrolizidine fragment in their structure. It should be noted that they provide a protective function for plants, making them poisonous [50]. However, these alkaloids can have useful medicinal properties, since they exhibit hepatotoxic, neurotoxic, genotoxic, and cytotoxic activities [51,52,53,54].

**Scheme 9:**
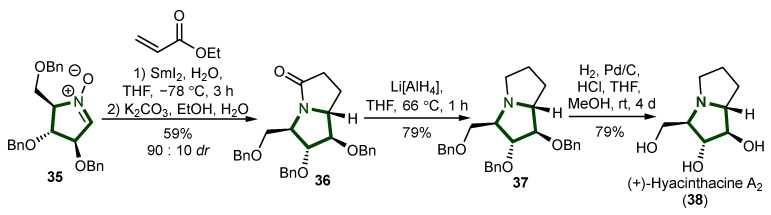
Synthesis of (+)-Hyacinthacine A_2_.

Pyrrolizidine alkaloids are predominantly synthesized from 3,4-dihydro-2*H*-pyrrole 1-oxide derivatives. Thus, in [55], a method for the synthesis of the alkaloid (+)-Hyacinthacine A_2_ (**38**) is described; it was isolated from the bulbs of *Muscari armeniacum* (Hyacinthaceae) and showed promising inhibitory activity against glycosidases [56,57]. Thus, nitrone **35** was treated with SmI_2_ and ethyl acrylate was added. Treatment of the reaction mixture with potassium carbonate in ethanol/water resulted in a lactam **36**. Reducing the lactam **36** using lithium aluminum hydride made it possible to obtain pyrrolizidine **37**. Then, the benzyl groups were removed, which made it possible to isolate (+)-Hyacinthacine A_2_ (Scheme 9).

**Scheme 10:**
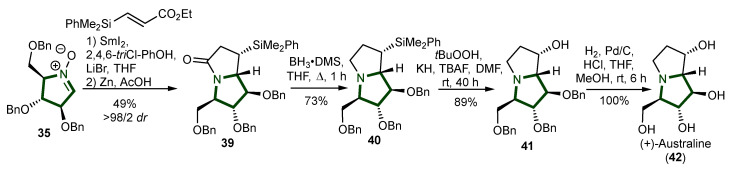
Synthesis of (+)-Australine.

**Scheme 11:**
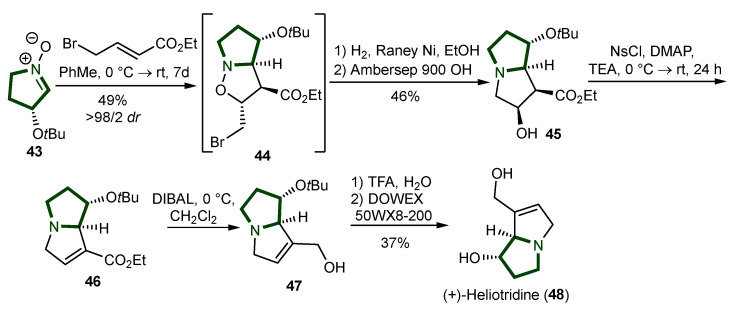
Synthesis of (+)-Heliotridine.

A similar strategy was used by P. Gilles and S. Py [58] to synthesize (+)-Australine (**42**), which was first isolated from the seeds of *Castanospmnum australe* [59]. This alkaloid is interesting because it inhibits amyloglucosidase and the breakdown of glycoproteins [60]. A recent review article [61] was devoted to the synthesis of Australine and its derivatives, which is indicative of the growing interest in this alkaloid. This alkaloid is capable of inhibiting amyloglucosidase and glycoproteins breakdown [60]. A recent review article [61]. The interaction of nitrone **35** with acrylate led to lactam **39** with a yield of 49% in the form of a single diastereomer. Then, through a series of stages, the target product **42** was isolated, and the last stage proceeded with quantitative yield (Scheme 10). We note the good yield and high enantioselectivity of the proposed reaction sequence.

(+)-Heliotridine (**48**) is a 1,2-unsaturated pyrrolizidine alkaloid which presents in plants of the Heliotropium genus [62,63]. Total synthesis of (+)-Heliotridine (**48**) was achieved by F.M. Cordero and colleagues [64]; their method is based on a highly selective 1,3-dipolar cycloaddition of (S)-3-tert-butoxypyrroline N-oxide **43** with ethyl 4-bromocrotone. The total yield of the product **48** was 17% (Scheme 11).

**Scheme 12:**
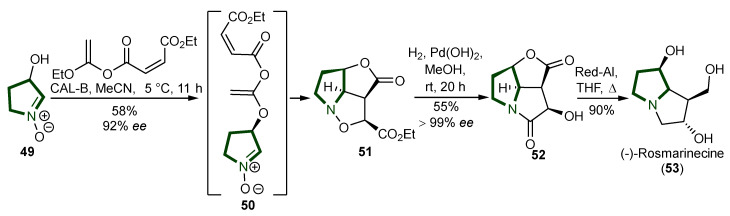
Synthesis of (-)-Rosmarinecine.

The asymmetric total synthesis of (-)-Rosmarinecine (**53**), first isolated in 1940 from the South African plant Senecio retrorsus [65], was carried out in three stages [66]. The key step was the reaction of nitrone **49** with an ester catalyzed by Candida antarctica lipase B (CAL-B). This was followed by intramolecular [3+2] dipolar cycloaddition, which resulted in a heterocycle **51** with an enantiomeric excess of 92%. Hydrogenation of the N–O bond and subsequent reduction with Red-Al (Sodium bis(2-methoxyethoxy)aluminum hydride) led to (-)-Rosmarinecine (**53**) (Scheme 12). The high enantioselectivity of this approach should be emphasized.

**Scheme 13:**
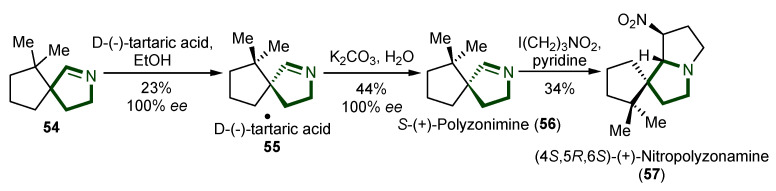
Synthesis of (+)-Nitropolyzonamine.

Nitropolyzonamine is one of the few alkaloids of animal origin. This unusual alkaloid, produced by the centipede Polyzonium rosalbum [67,68], protects the organism from enemies. The authors of [69] describe a method used to synthesize (4S,5R,6S)-(+)-Nitropolyzonamine (**57**). The starting compound is an enantiomerically pure imine **56**, which was obtained from heterocycle **54** by treatment with D-(-)-tartaric acid. Conversion of the salt to the free base allowed the enantiospecific isolation of S-(+)-Polyzonimine (**56**) with quantitative yield, which reacted with 3-iodo-1-nitropropane to form (4S,5R,6S)-(+)-Nitropolyzonamine (Scheme 13). The proposed approach benefits from its short synthetic scheme (3 stages), high enantioselectivity and use of readily available D-(-)-tartaric acid as both a reagent and a catalyst.

## 4. Synthesis Quinolizidine Alkaloids

**Scheme 14:**
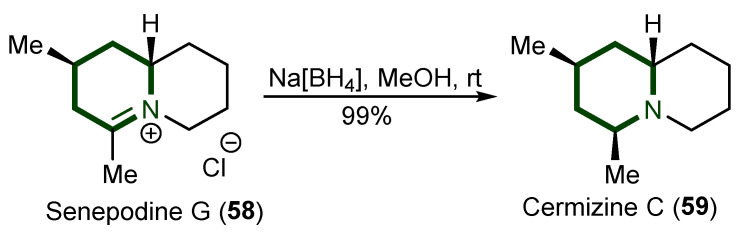
Synthesis of (-)-Cermizine C.

There are more than 200 known quinolizidine alkaloids, which are united by the presence of a 1-azabicyclo[4.4.0]decane core [70,71]. Like most alkaloids, quinolizidine alkaloids protect plants from pests and diseases, i.e., they perform a protective function. Despite this, many representatives of this class of alkaloids are utilized by humans [72]. Reduction of (-)-Senepodine G (**58**) with sodium borohydride in methanol resulted in (-)-Cermizine C (**59**) [73] (Scheme 14). This alkaloid is found in plants of the genus *Lycopodium* [74].

**Scheme 15:**
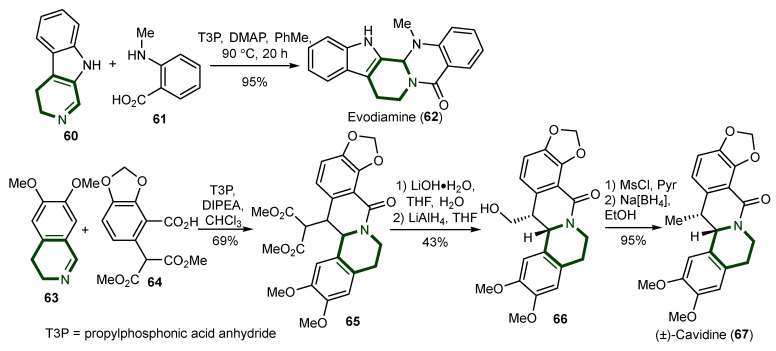
Synthesis of Evodiamine and Cavidine.

The work [75] describes a method used for the synthesis of Evodiamine (**62**) from dihydrocarboline **60** and anthranilic acid **61** (Scheme 15). The great potential of evodiamine as a new drug is described in sufficient detail in review articles that summarize the biological activity of this alkaloid [76,77,78,79,80]. The same work [75] describes the method used to synthesize an equally important alkaloid, Cavidine (**67**). Thus, Cavidine exhibits various biological activities, such as antiulcer [81] and anti-inflammatory effects [82], and improves lung function [83]. In the first step, the reaction of imine **63** with an acid produced ester **65**, which was hydrolyzed and decarboxylated using lithium hydroxide in aqueous THF, followed by reduction with lithium aluminum hydride, resulting in alcohol **66** as a single diastereoisomer. Mesylation followed by deoxygenation resulted in (±)-Cavidine.

**Scheme 16:**
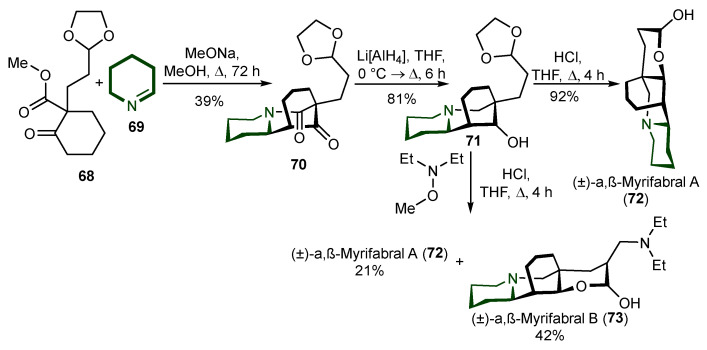
Synthesis of (±)-α,β-Myrifabral A and B.

In [84], acetal **69** and 2,3,4,5-tetrahydropyridine **69** were used as starting compounds for the preparation of alkaloids (±)-α,β-Myrifabral A (**72**) and B (**73**), the reaction of which resulted in lactam **70**. Reducing lactam **70** with lithium aluminum hydride led to the formation of amino alcohol **71** in the form of a mixture of two diastereomers; however, via column chromatography, it was possible to isolate the main diastereomer with 81% yield. Treating alcohol **71** with hydrochloric acid promoted cyclization, leading to the formation of (±)-α,β-Myrifabral A (**72**) at 92% yield. It should be noted that synthesis is possible on a scale of several grams. Treating acetal **71** with hydrochloric acid and *N*,*N*-diethyl-*O*-methylhydroxylamine led to a mixture of (±)-α,β-Myrifabral A and B, which were successfully separated (Scheme 16). It should be noted that the Myrifabral alkaloids are a relatively new, small family of polycyclic alkaloids. These alkaloids were first isolated in 2002 from the plant of *Myrioneuron nutans* species [85,86], and are currently being actively studied.

**Scheme 17:**
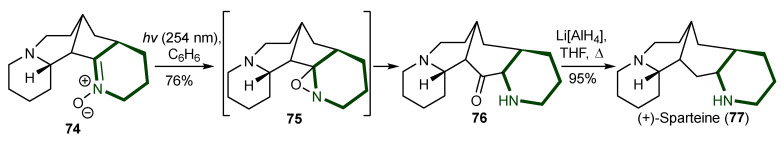
Synthesis of (+)-Sparteine.

The work [87] describes the enantioselective synthesis of the alkaloid (+)-Sparteine (**77**), based on the Beckmann photo-rearrangement (Scheme 17). Photolysis in benzene at 254 nm provided a smooth rearrangement of lactam **76** with a yield of 76%. Removal of the lactam carbonyl using lithium aluminum hydride led to (+)-Sparteine in a virtually quantitative yield. The key photochemical stage proceeds in mild conditions without any metal catalysts and provides entry to the rather complex scaffold. The operational simplicity and readily available reagents render photochemical transformations very promising for industrial applications. It should be noted that Sparteine is one of the few alkaloids that is currently used in medical practice as an antiarrhythmic agent and sodium channel blocker. In addition, Sparteine is widely used in asymmetric synthesis as a chiral ligand [88,89], which makes this alkaloid a very valuable product.

## 5. Synthesis of Aspidosperma Alkaloids

**Scheme 18:**
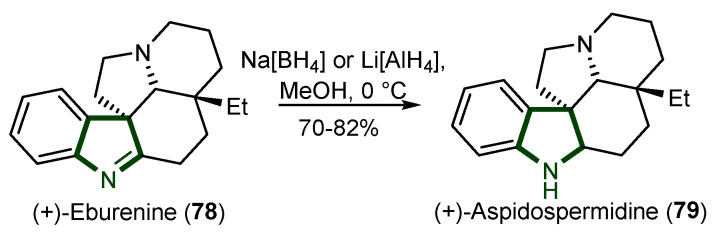
Synthesis of (+)-Aspidospermidine.

Aspidosperma alkaloids are a large family of naturally occurring and structurally related indole alkaloids with diverse biological activities [90,91]. Therefore, this important class of compounds has been the subject of review articles related to their synthesis [92,93,94]. Aspidospermidine is the simplest representative of this class of alkaloids, from which more complex structural alkaloids are synthesized. Notably, plants were the first source of bioactive compounds due to their abundance, diversity and availability. Thus, the semi-synthetic drugs often have better availability. There are a number of advantages associated with semi-synthetic alkaloid-based drugs, including a short synthesis scheme, a high yield of optically pure product, increased bioavailability, improved solubility, decreased toxicity, and increased selectivity [95,96]. Aspidospermidine (**79**) was synthesized quite easily from another alkaloid, Eburenine (**78**), by reduction with sodium borohydride or lithium aluminum hydride [97,98,99,100,101,102,103,104] (Scheme 18). The alkaloid Eburenine (**78**) is a major component of *Rhazya stricta* [105,106].

**Scheme 19:**
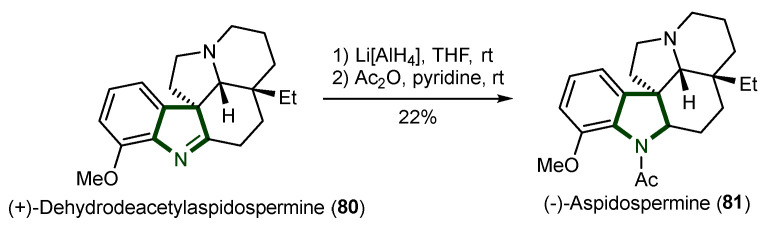
Synthesis of (-)-Aspidospermine.

Treatment of imine **80** lithium aluminum hydride and acetic anhydride/pyridine led to the production of a mild form of (-)-Aspidospermine (**81**) [99,107] (Scheme 19). Aspidospermine was first isolated from a tree of the genus Aspidosperma. Aspidospermine was found to possess adrenergic blocking activities for a variety of urogenital tissues [108], antimalarial activity [109], etc. Dehydrodeacetylaspidospermine (**80**) is a component of plants of the genus *Winchia* [110].

**Scheme 20:**
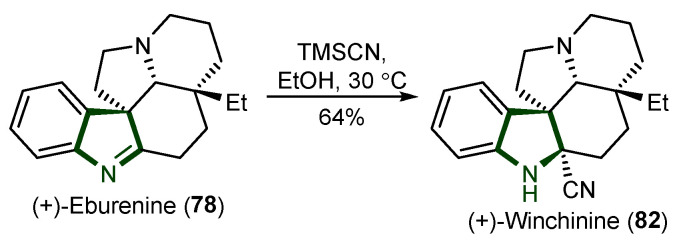
Synthesis of (+)-Winchinine.

The addition of trimethylsilyl cyanide to imine **78** in ethanol allowed the synthesis of (+)-Winchinine B (**82**), which was first isolated from the leaves of *Winchia calophylla* (Scheme 20) [111,112]. High enantioselectivity in the absence of any chiral catalysts is the salient feature of this reaction.

**Scheme 21:**
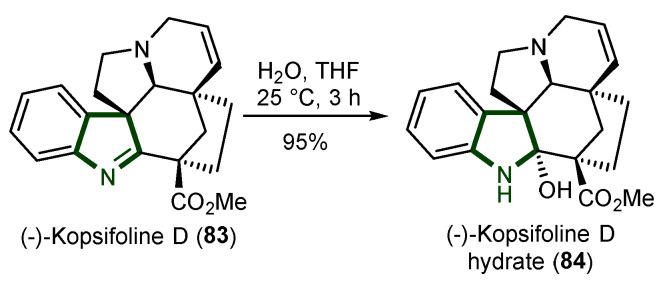
Synthesis of (-)-Kopsifoline D hydrate.

Addition of water to (-)-Kopsifoline D (**83**) yielded (-)-Kopsifoline D hydrate (**84**) (Scheme 21). It should be noted that a synthesis method consisting of simply stirring the imine **83** in a tetrahydrofuran–water mixture allowed the product to be isolated at 95% yield [113].

**Scheme 22:**
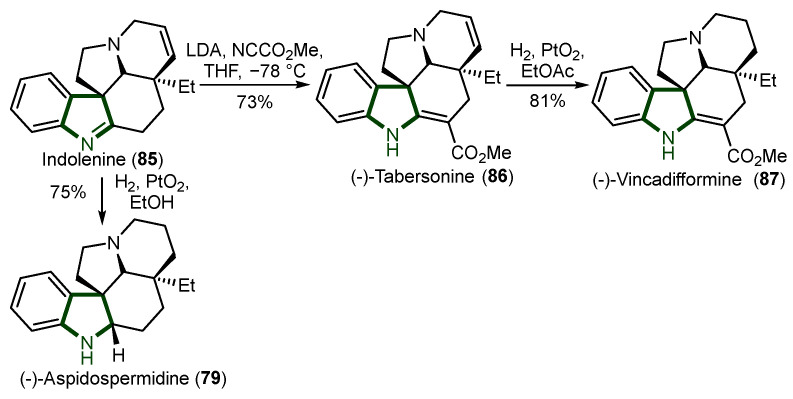
Synthesis of (−)-Tabersonine, (−)-Vincadifformine, and (−)-Aspidospermidine.

In a series of papers [98,114,115,116], enantioselective synthesis of the alkaloids (−)-Tabersonine (**86**), (−)-Vincadifformine (**87**), and (−)-Aspidospermidine (**79**) (Scheme 22) is described. Indolenine (**85**) serves as a key intermediate product in the synthesis of Aspidosperma alkaloids. Complete hydrogenation of Indolenine (**85**) in the presence of Adams catalyst in ethanol at room temperature led to (−)-Aspidospermidine with a yield of 75%. From indolenine, it is also possible to synthesize (−)-Tabersonine and (−)-Vincadifformine, with product yields exceeding 70%. Here, we would like to mention the alkaloid (−)-Tabersonine, since it has a variety of biological activities: it suppresses the formation of amyloid fibrils [117], attenuates kidney damage caused by obesity [118], and exhibits antitumor [119,120] and anti-inflammatory [121] activities.

**Scheme 23:**
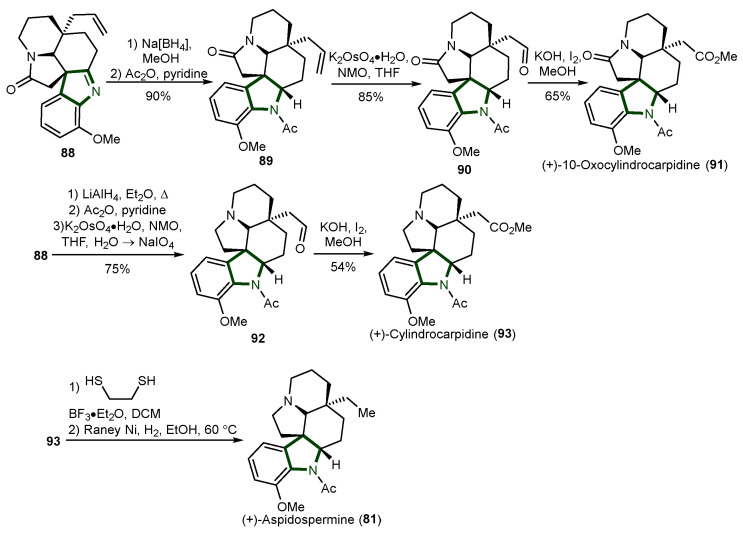
Synthesis of (+)-10-Oxocylindrocarpidine, (+)-Cylindrocarpidine, and (+)-Aspidospermine.

An original enantioselective synthetic route has been proposed for the preparation of the alkaloids (+)-10-Oxocylindrocarpidine (**91**), (+)-Cylindrocarpidine (**93**), and (+)-Aspidospermine (**81**) [122]. The key steps of the approach are reduction to the free amine and its acylation to compound **88**. For the synthesis of (+)-10-Oxocylindrocarpidine, the alkene was oxidized with potassium osmate to aldehyde **90** in 85% yield. Subsequent oxidation of the aldehyde group with I_2_ in the presence of potassium hydroxide produced the target molecule **91**. Oxidation of compound **92** under the same conditions produced (+)-Cylindrocarpidine (**93**). Using a two-step transformation involving mercaptalization followed by hydrogenation with Raney nickel, (+)-Aspidospermine (**81**) was synthesized (Scheme 23).

**Scheme 24:**
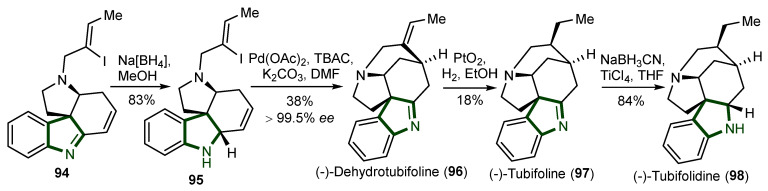
Synthesis of (-)-Tubifolidin.

The reduction of an imine to an amine is described; this amine, in turn, underwent an intramolecular Heck reaction to form (-)-Dehydrotubifolin (**96**). (-)-Tubifolin (**97**) was synthesized by selective hydrogenation of the double bond in (-)-Dehydrotubifolin (**96**), and subsequent reduction of the imine group led to the formation of (-)-Tubifolidin (**98**) (Scheme 24) [123,124].

**Scheme 25:**
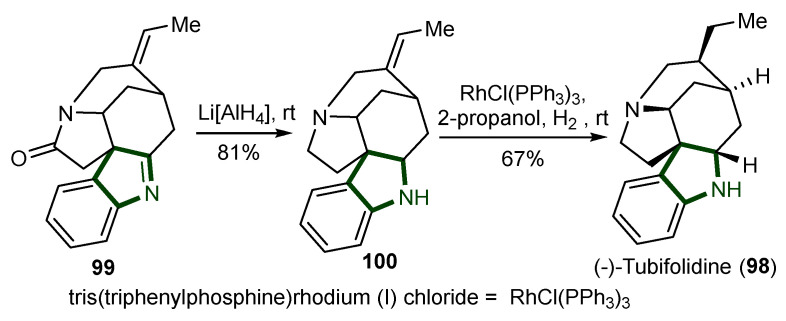
Synthesis of (-)-Tubifolidin.

Reduction of compound **99** with lithium aluminum hydride in tetrahydrofuran resulted in the formation of amine **100**. Amine **100** was finally converted to Tubifolidin (**98**) in the presence of tris(triphenylphosphine) rhodium (I) chloride in benzene and 2-propanol at room temperature at 67% yield (Scheme 25) [125].

**Scheme 26:**
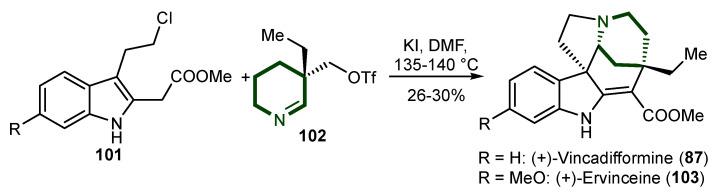
Synthesis of (+)-Vincadifformine and (+)-Ervinceine.

An asymmetric total synthesis of (+)-Vincadifformine (**87**) and (+)-Ervinceine (**103**) is described in [126,127]. Thus, indole **101** reacted with imine **102** in the presence of potassium iodide in DMF to form alkaloids (Scheme 26). Interestingly, the authors successfully isolated an enantiomerically pure compound without use of chiral catalysts. Moreover, the reaction was carried out at elevated temperature, which is not typical for asymmetric synthesis.

**Scheme 27:**
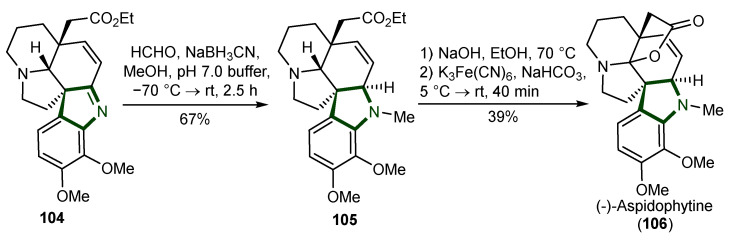
Synthesis of (-)-Aspidophytine.

The stereoselective reduction of the imine 104 followed by methylation to yield polycycle 106 deserves special attention [128,129]. Saponification of the ester 105 and subsequent subjection of the resulting carboxylic acid to oxidative lactone formation conditions resulted in the formation of (-)-Aspidophytine (106) (Scheme 27). (-)-Aspidophytine (106) was first obtained from *Haplophyton crooksii*, or the common cockroach plant, which is native to North America [130]. Components of this plant have been used for thousands of years to control cockroaches. The mechanism of action is probably related to the inhibition of acetylcholinesterase [131].

## 6. Synthesis of Steroidal Alkaloids

Steroid alkaloids, as the name suggests, have a steroid skeleton with corresponding functional groups. Steroid alkaloids exhibit a wide range of biological activities, including chemotherapeutic [132,133,134], antiviral [135], and antimicrobial [136] activity, and can be used to treat osteoporosis [137].

**Scheme 28:**
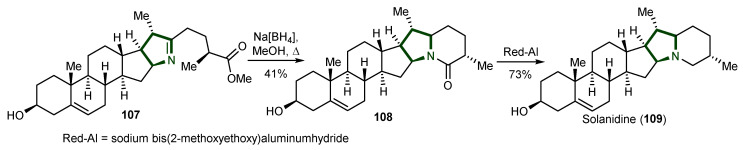
Synthesis of Solanidine.

For the synthesis of Solanidine (**109**), polycycle **107** was used as a starting compound, which was sequentially reduced with sodium borohydride and Red-Al [138] (Scheme 28). This alkaloid is interesting because it is a very important precursor for the synthesis of hormones and some pharmacologically active compounds, such as progesterone and cortisone [139]. Solanidine is a poisonous alkaloid that is found in fairly large quantities in plants of the *Solanaceae* family. This has made Solanidine a precursor for the further synthesis of useful compounds. Solanidine itself is a biomarker for various cytochrome CYP2D6 genes [140,141].

**Scheme 29:**
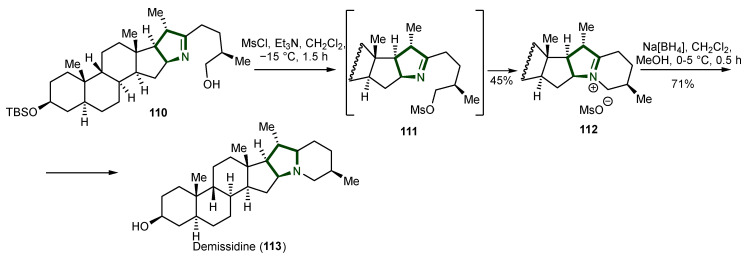
Synthesis of Demissidine.

The synthesis of Demissidine (**113**) is described in [142]. Thus, steroid **110** reacted with mesyl chloride to form compound **111**, which spontaneously cyclized to iminium salt **112** in the presence of triethylamine (Scheme 29); compound **112** easily transformed the target compound **113** in the presence of sodium borohydride.

## 7. Synthesis of Other Alkaloids

**Scheme 30:**
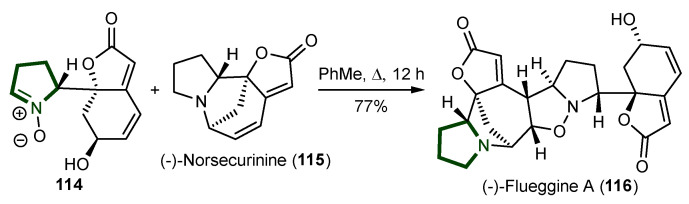
Synthesis of (-)-Flueggine A.

This section contains a description of the syntheses of alkaloids that were not included in the previous sections. (-)-Flueggine A (**116**) was isolated from the leaves of *Flueggea virosa* in 2011 [143]. The leaves of this plant are used in traditional Chinese medicine to treat skin diseases, and this alkaloid is likely responsible for the effectiveness of the treatment [144]. In [145,146] the synthesis of (-)-Norsecurinine (**115**) based on the 1,3-dipolar addition of compound **115** in boiling toluene for 12 hours is described (Scheme 30).

**Scheme 31:**
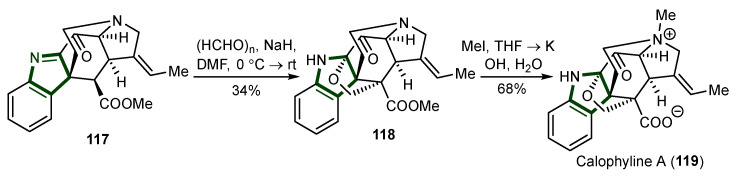
Synthesis of Calofilin A.

Calofilin A (**119**) was first isolated in 2012 from *Winchia calophylla* [147]. Since this nitrogen- and oxygen-containing alkaloid contains many different functional groups and several fused rings, complete synthesis was carried out for the first time in 2016 under the guidance of Professor Liansuo Zu [148]. Calofilin A (**119**) could be synthesized in two stages from imine (Scheme 31). Imine **117** reacted with formaldehyde in the presence of sodium hydride as a base and dimethylformamide as a solvent to form compound **118** in 34% yield. Finally, by *N*-methylation and saponification, it was possible to obtain the natural product Calofilin A (**119**) [149].

**Scheme 32:**
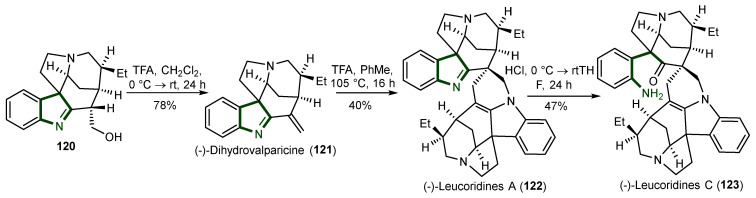
Synthesis of (-)-Leucoridins A and C.

In 2010, Kam and co-workers isolated (-)-Leucoridins A (**122**) and C (**123**) from the bark of *Leuconotis griffithii* [150]. The authors of [151,152] proposed a fairly effective method for the synthesis of these complex alkaloids. In the first stage, alcohol **120** was dehydrated with trifluoroacetic acid, which led to the formation of (-)-Dihydrovalparicin (**121**). Replacing the solvent with toluene and boiling for 16 h led to the formation of (-)-Leucoridin A (**122**) at 40% yield. The addition of hydrochloric acid to (-)-Leucoridine A (**122**) resulted in the opening of the indolenine ring to form (-)-Leucoridine C (**123**) at 47% yield (Scheme 32).

## 8. Conclusions

In conclusion, we note that the development of new methods for the synthesis of alkaloids is of great practical importance in the search for new drugs of plant origin. Since the alkaloid content in plant materials can be extremely low, synthetic methods will make it possible to obtain these valuable products in sufficient quantities. The emergence of drug resistance is also important, so the development of effective synthesis methods is becoming increasingly important.

Analysis of data from the literature indicates that imines are successfully involved in the synthesis of not only relatively simple alkaloids of the pyrrolizidine and indolizidine series, but also of a large group of structurally complex Aspidosperma alkaloids. This is primarily due to the wide variety of types of reactions into which cyclic imines can participate. First of all, they may be reduced to amines under mild conditions, with almost quantitative yield of the product, which in turn participate in further reactions.

Reactions of imines with nucleophiles are caused by the carbon atom; they are very diverse and are widely used in the synthesis of alkaloids. This is a key step for the synthesis of alkaloids such as (+)-Winchinine B, (-)-Kopsifoline D hydrate. Alkylation of the nitrogen atom leads to iminium salts, which ultimately makes it possible to obtain (-)-Swainsonine, Serratezomine A, and (-)-Isoschizogamine. Imines undergo various cycloaddition reactions, which lead to the formation of (-)-Rosmarinecine, (±)-α,β-Myrifabral A, (-)-Norsecurinine, and (+)-Heliotridine. Imines undergo ring opening much less frequently, so this process makes it possible to synthesize the alkaloid (-)-Leucoridine C, which has a complex structure.

A feature of cyclic imines is their ability to react under mild conditions. These conditions play a key role in preventing racemization of the precursor, which is a very big problem in multi-step syntheses of optically pure compounds.

However, the inevitable drawback of the imine-based approaches is the hydrolytic instability of most of imines, which implies strictly anhydrous conditions, hindering their syntheses and increasing their costs.

Photochemical transformations are among the most promising “green chemistry” approaches for the synthesis of natural products starting from imines. Nonetheless, only the photochemical reaction of octahydro-2*H*-quinolizines is known so far, which has been applied to the synthesis of a sparteine. Thus, further studies in this field are of great interest.

The data presented in this review indicate that, every year, dozens of new alkaloids are isolated from plants, which are later synthesized by chemists in laboratory settings. Therefore, the chemistry of cyclic imines is currently actively developing and is a popular area of modern organic and medicinal chemistry.
